# POSS-Functionalized Graphene Oxide/PVDF Electrospun Membranes for Complete Arsenic Removal Using Membrane Distillation

**DOI:** 10.1021/acsapm.0c01402

**Published:** 2021-03-05

**Authors:** Sebastian Leaper, Edgardo Oscar Avendaño Cáceres, Jose Miguel Luque-Alled, Sarah H. Cartmell, Patricia Gorgojo

**Affiliations:** † Department of Chemical Engineering and Analytical Science, School of Engineering, The University of Manchester, Manchester M13 9PL, UK; ‡ Faculty of Engineering, Universidad Nacional Jorge Grohmann, Avenida Miraflores S/N, Miraflores 23000, Peru; § Department of Materials, School of Natural Sciences, The University of Manchester, Manchester M13 9PL, UK

**Keywords:** graphene oxide, electrospun, membrane distillation, arsenic removal, POSS, nanocomposite membrane

## Abstract

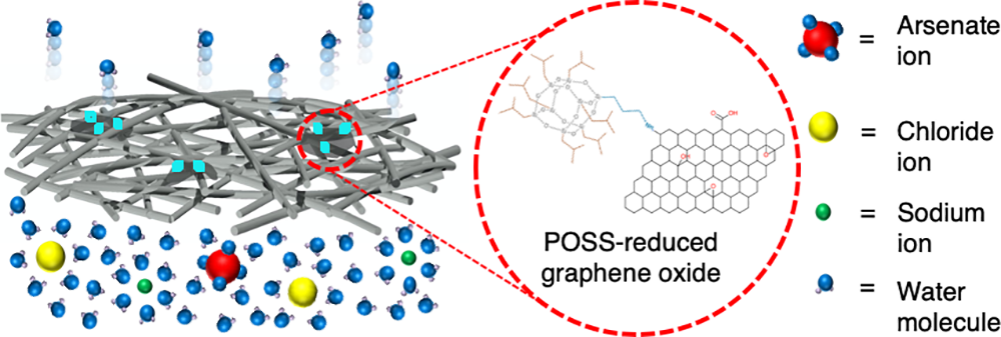

This work demonstrates very high removal rates (below the detection limit of 0.045 ppb) of inorganic arsenic from water using electrospun polyvinylidene difluoride (PVDF) membranes enhanced by the addition of functionalized graphene oxide in membrane distillation. This shows potential for applications in the many parts of the world suffering from arsenic-contaminated groundwater. These membranes were enhanced by the addition of reduced graphene oxide functionalized with superhydrophobic polyhedral oligomeric silsesquioxane molecules (POSS-rGO) into the spinning solutions. The flux of the best-performing rGO-enhanced membrane (containing 2 wt % POSS-rGO) was 21.5% higher than that of the pure PVDF membrane and almost double that of a commercial polytetrafluoroethylene (PTFE) membrane after 24 h of testing, with rejection values exceeding 99.9%. Furthermore, the flux of this membrane was stable over 5 days (∼28 L m^–2^ h^–1^) of continuous testing and was more stable than those of the PTFE and control membranes when treating a concentrated fouling solution of calcium carbonate and iron(III) sulfate heptahydrate. It also achieved higher permeate quality in these conditions. The Young’s modulus and ultimate tensile strength of the best-performing membrane increased by 38 and 271%, respectively, compared to the pure polymer membrane, while both had similar porosities of ∼91%.

## Introduction

Arsenic contamination in groundwater is a growing problem, threatening drinking water supplies in many parts of the world. Locations such as the Ganga River Basin, encompassing considerable parts of India, Bangladesh, Nepal, and Tibet;^1^ the Amazon Basin, including regions of Brazil and Peru;^2^ and the Datong Basin in North West China^3^ are but a few of the many areas that are experiencing dangerously high (>10 ppb^4^) levels of arsenic in the groundwater. Arsenic can exist in four valence states: −3, 0, +3, and +5, with inorganic As^3+^ and As^5+^ being the most common and relevant to groundwater contamination. Long-term exposure to these forms of arsenic increase risks of skin, lung, bladder, and kidney cancer as well as hypertension and cardiovascular disease, among others. Ingesting large doses of inorganic arsenic results in gastrointestinal symptoms, disruptions to cardiovascular and nervous system functions, and eventually death.^5^ There are a variety of both natural and man-made sources of arsenic in the environment, and both must be monitored in order to mitigate risk to human health.

Various techniques for removing inorganic arsenic from groundwater exist, including oxidation, coagulation-flocculation, adsorption, ion exchange, and membrane filtration. However, these can suffer from low separation efficiency or require complex multistep processes in order to operate effectively over time. Membrane distillation (MD) is a simple and robust technology for achieving very high removal rates of dissolved inorganic substances.^6^ By utilizing nonwetted hydrophobic membranes to separate heated feed water from the permeate collection stream, membrane distillation allows the passage of vapor through the membrane while keeping all dissolved inorganic substances in the feed water.^7^ This system is able to treat highly concentrated water because it operates on a gradient in vapor pressure rather than hydraulic pressure like reverse osmosis and is therefore able to treat brines toward and even beyond saturation. This is an advantage in the case of arsenic removal since it can enable the complete recovery of water for zero liquid discharge applications.^8,9^

Much research effort has been put toward achieving higher production rates and lower propensity for pore wetting in MD in order for it to compete with other technologies.^10,11^ Designing high-performance membranes is one of the main ways of achieving this. Over the years, it seems that phase inversion has all but given way to electrospinning as the most effective way to fabricate MD membranes (although the cost-effectiveness and long-term performance stability have not yet been proven). By producing a network of randomly aligned polymer fibers, it is possible to obtain membranes with very high porosity and highly interconnected pores, which have been applied to applications ranging from tissue engineering,^12^ energy storage,^13^ air filtration,^14^ and others.^15−17^ In order to further enhance these properties, it is possible to incorporate nanomaterials into the polymer solutions prior to spinning as has been shown previously.^18−20^ In this work, graphene oxide (GO) was functionalized with superhydrophobic polyhedral oligomeric silsesquioxane (POSS) molecules, which have a cage-like structure composed of silicon and oxygen atoms and represent the smallest possible silica nanoparticle size. This organic–inorganic hybrid molecule has been shown to improve the thermal stability and mechanical properties of polymer composites due to strong hydrophobic interactions with the matrix^21^ and has been covalently grafted onto graphene oxide to induce superhydrophobic properties.^22^ While most publications on graphene-based membranes utilize stacked sheets of GO to form a network of nanosized pores,^23^ here, the high strength and tailorable surface of GO have been exploited and utilized as a nanoadditive. In order to maximize the performance in MD, membrane porosity and hydrophobicity must be maximized. However, as porosity increases, mechanical strength decreases and a balance must be struck. By functionalizing GO with POSS, thermally reducing it, and blending it with the PVDF polymer, the aim was to simultaneously improve the mechanical properties of the electrospun membranes, thereby increasing the durability, allowing for porosities exceeding 90%, increasing the hydrophobicity, reducing the chance of pore wetting, and increasing the quality of the MD permeate. The efficacy of this approach was tested by using the membranes to treat saline arsenic-contaminated groundwater, simulating that from the Tacna region in Peru, using air gap membrane distillation – the most thermally efficient variant of membrane distillation.^24^ Inorganic fouling studies were also conducted using highly concentrated calcium carbonate and iron(III) sulfate heptahydrate solutions to test the stability of the process in these harsh conditions. Calcium carbonate, iron oxides, and calcium sulfate are well-known inorganic foulants in MD.^25−28^

## Results and Discussion

### Functionalized Graphene Oxide Characterization

Functionalization graphene oxide was analyzed by X-ray photoelectron spectroscopy. POSS-GO and POSS-rGO surveys expectedly show the presence of silicon and nitrogen as a consequence of the GO functionalization. The C1s high-resolution spectra of both graphene derivative materials were acquired in order to investigate the chemical modifications in detail and are presented in Figure S1. The C1s spectrum of POSS-GO exhibits a doublet, which was broken down into four peaks. The peaks were assigned to the C–C, C–N, C–O, and C=O bonds present in POSS-GO as a consequence of the siloxane grafting. According to the molecular structure of the siloxane, C–Si bonds (observed at 284 eV^29,30^) are expected. However, this peak is not suggested by the software in any case, possibly due to the presence of a huge peak for C–C, which makes the other peaks next to it negligible.^22,31^ Besides, a new band corresponding to C–N is observed around 285.6 eV.^31−34^ In addition, the peaks corresponding to C–O and C=O are decreased in POSS-GO as a consequence of the reaction between the epoxy and carboxylic groups with the amine group in POSS.^22,31^ The C1s spectrum of POSS-rGO shows a decrease in C–O in comparison to POSS-GO, which confirms the reduction of the GO. The degree of reduction can also be observed in the increase of the C/O ratio (3.01 for POSS-GO and 3.64 for POSS-rGO, calculated from the percentage values shown in Table S1).

**Figure 1:**
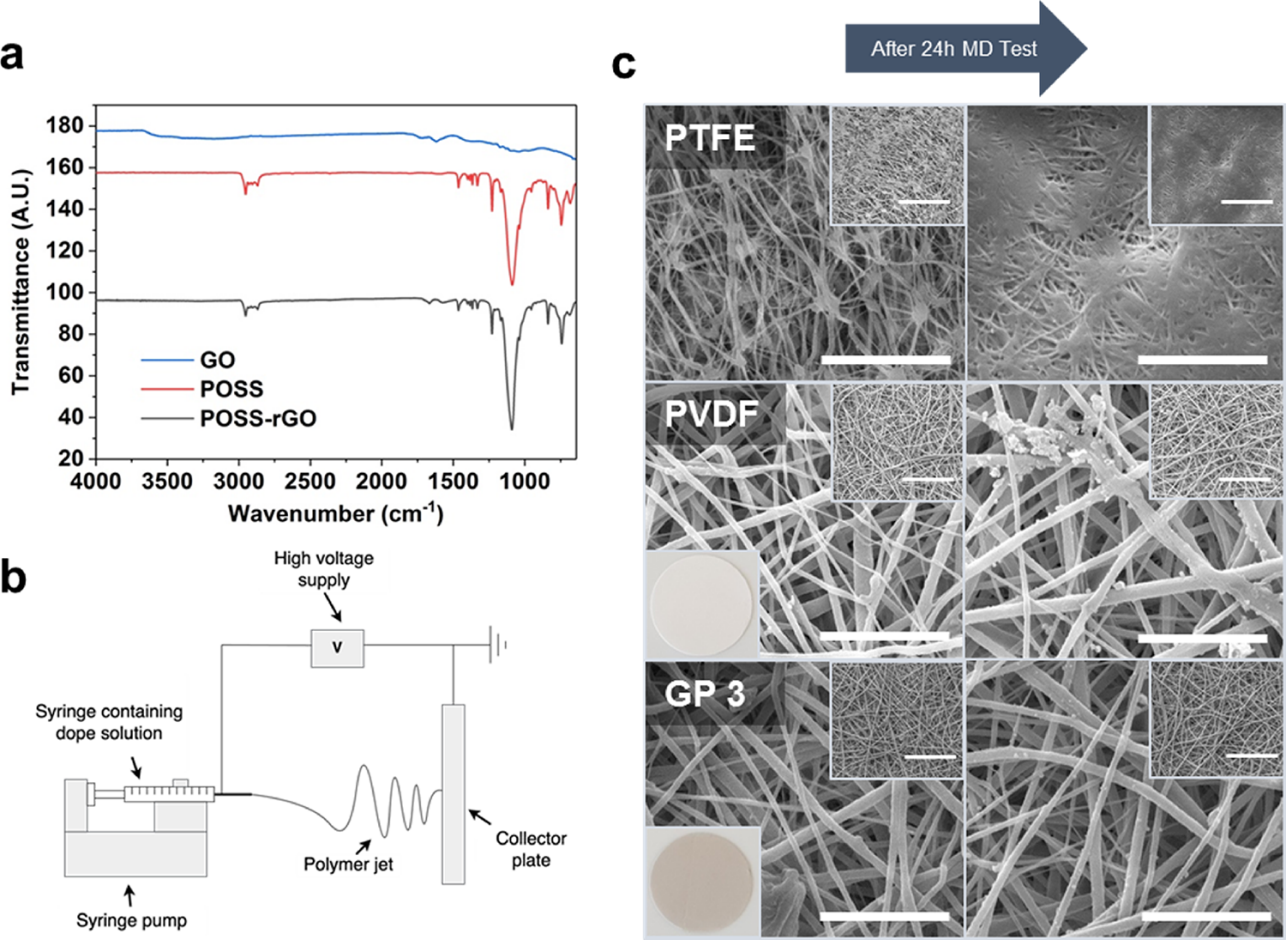
(a) Chemical analysis of graphene oxide, POSS, and POSS-functionalized graphene oxide using Fourier transform infrared spectroscopy; (b) schematic of the electrospinning setup used to fabricate the membranes; (c) SEM images of the commercial PTFE membrane (top), the unmodified electrospun PVDF membrane (middle), and the modified electrospun membrane containing 3 wt % POSS-rGO (bottom). The right hand column shows the same membranes after 24 h of testing in MD, and inset images in the top right corners are the same membranes taken at lower magnification. For the PTFE images, the magnifications are 12,000× and 6000× (inset) with scale bars representing 5 and 20 μm, respectively. For the PVDF and GP 3 membranes, the magnifications are 3000× and 800× (inset) with scale bars representing 10 and 50 μm, respectively. Inset images in the bottom left corners of the PVDF and GP 3 micrographs are photographs of the membranes cut into disks 2 cm in diameter and placed on the same white background.

In addition, the chemical functionalization of GO was investigated using ATR-FTIR, as presented in Figure 1a. For the GO, characteristic peaks are found at 1090, 1248, 1600, and 1750 cm^–1^, which originate from alkoxy CO stretching vibrations, epoxy CO stretching vibrations, CC stretching in the aromatic ring, and CO carboxyl stretching, respectively. Meanwhile, the broad band from 2990 to 3600 cm^–1^ is attributed to OH stretching.^35^ The spectrum is modified considerably for POSS-rGO (functionalized at 80 °C), which shows a pronounced peak at 1110 cm^–1^ as a result of Si–O–Si stretching. In addition, a weaker band in the range of 2750–3000 cm^–1^, attributed to the isobutyl groups, which extend from the POSS molecule, and the peak at 1650 cm^–1^, corresponding to the C=O stretch of the amide (which is absent from POSS), show the successful grafting of this functional molecule onto graphene oxide.^22^

### Electrospun Membrane Characterization

Membranes were fabricated with concentrations of GO-based fillers in the electrospinning solution ranging from 0.5 to 3 wt %. Prepared membranes were labeled as GP followed by the wt % used, so for instance, GP 3 is a membrane prepared from a dope solution containing 3 wt % POSS-rGO. The components of each dope solution are summarized in Table S2. The high optical absorbance of the POSS-rGO resulted in dark black dope solutions, even at the lowest loading of 0.5 wt %. However, when the polymer solidified during the electrospinning process, the appearance altered from transparent to white, occluding the dark color of the POSS-rGO and resulting in a light gray shade. This can be seen in the optical photographs shown as inset images in Figure 1c. EDX images (Figure S2) also show a pronounced Si peak for the GP 2 membrane, corresponding to POSS-rGO. In the elemental map, there are purple (Si) clusters concentrated on the surface of graphene flakes, as highlighted by red dashed circles. This is further evidence of successful grafting of POSS molecules onto the graphene as well as successful incorporation of POSS-rGO into the electrospun membranes.

Figure 1c also shows the scanning electron microscopy (SEM) images of the unmodified (PVDF), modified (GP 2) electrospun membranes, and the commercial PTFE membrane before and after MD testing. As can be seen, the electrospun membranes composed of randomly aligned fibers containing very little, if any, beading, indicating that the addition of POSS-rGO did not affect the fiber stability during spinning. The images show fairly little evidence of fouling, which is expected given the high solubility of NaCl. More SEM images can be found in Figure S3. Small crystals do appear to have precipitated on the fibers upon drying of the membranes but are not large enough to block the pores completely. However, the image of the PTFE membrane (Figure 1c – top) whose magnification was 12,000× (compared to 3000× for the electrospun membranes) does show some evidence of pore blocking. This may be related to the fact that the pores of the PTFE membrane are 1–2 orders of magnitude smaller than the electrospun membranes, meaning that even small crystals are capable of completely blocking the pores. This may be another advantage of electrospun membranes. Their larger pore size requires foulant crystals to grow to sufficient size in order to block them. Utilizing process conditions, which prevent crystal growth to such sizes, may be an effective strategy to control fouling during the treatment of highly concentrated brines. This is discussed in more detail later.

**Figure 2:**
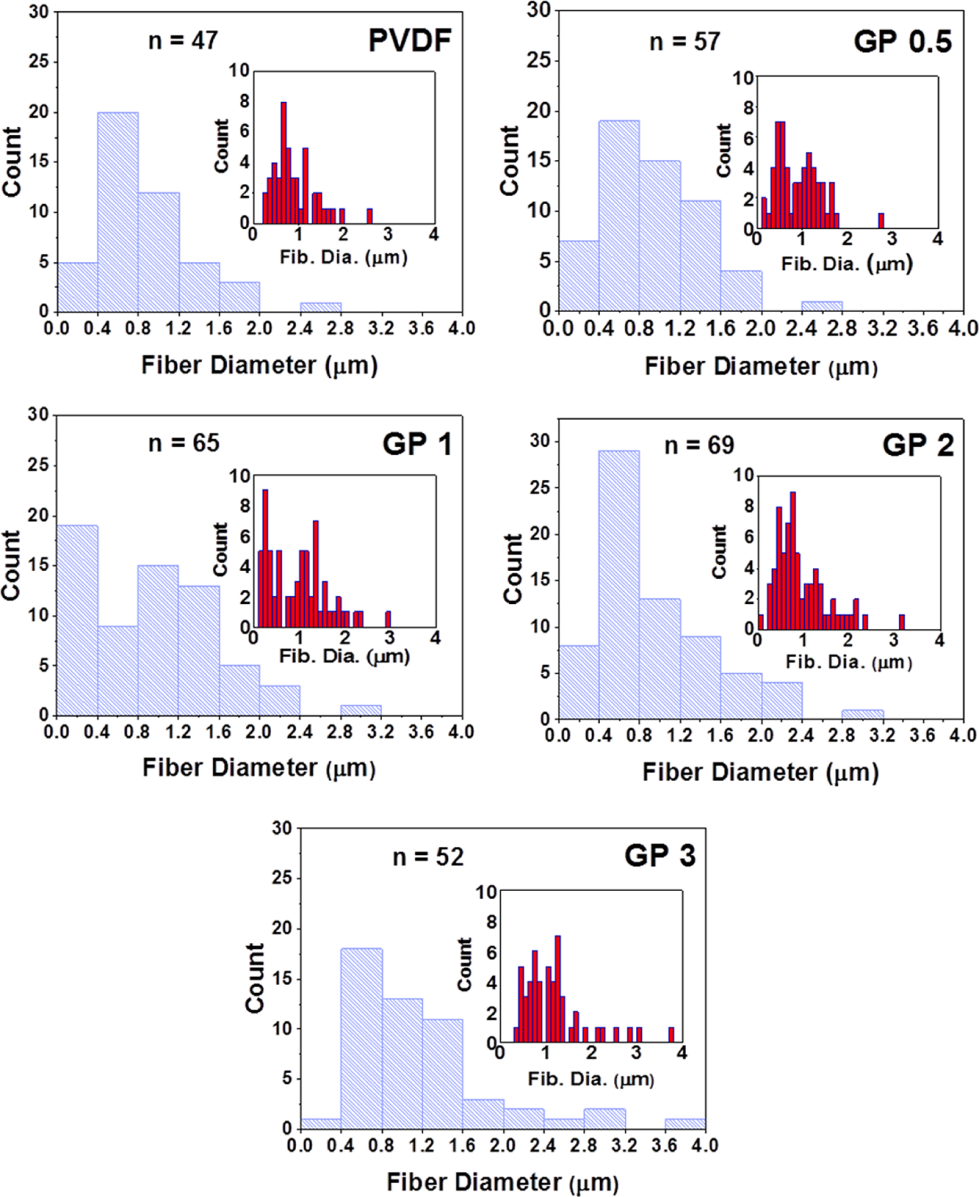
Histograms representing the size distributions of the electrospun fibers as measured from SEM images, where *n* is the number of measurements taken. The blue bars represent a bin width of 0.4 μm, and the red bars (inset) represent a bin width of 0.1 μm for a more fine-grained breakdown of the fiber size distribution.

Figure 2 contains histograms showing the size distributions for the nanofibers. In all but one case, the most populated size range was between 400 and 800 nm. The exception was GP 1, which had more nanofiber diameters in the range of 0–400 nm. The membrane with the largest range of fiber sizes was GP 3, which had fibers as large as 4 μm. This may be the result of partial agglomeration of the GO-based sheets affecting the balance between the electrostatic and viscoelastic forces during the electrospinning process. More specifically, agglomeration could result in local increases in the solution viscosity, which acts against the compressive electrostatic forces from the needle as well as the tensile force caused by the whipping of the fiber, resulting in a larger fiber being produced.^36^ More evidence of the effect of rGO in the polymer solution is found in the red histograms, which have a smaller bin size of 100 nm. While the proportion of nanofibers under 800 nm in diameter is similar (40–50%) for the electrospun membranes (excluding GP 3), there are significant differences in the number of nanofibers under 300 nm. For pure PVDF, this proportion was just 4% compared with 12, 28, and 12% for GP 0.5, GP 1, and GP 2, respectively. This higher number of very small fibers for the POSS-rGO membranes can be observed when looking carefully at the SEM images. This is possibly the result of the conductivity of POSS-rGO increasing the electrostatic repulsion experienced by the fiber as it travels to the collector, resulting in thinner fibers. A deviation from this trend is again seen in GP 3 for which only 2% of fibers measured were below 300 nm in size. This is possibly due to the increased solution viscosity and agglomeration of the graphene, as has been observed elsewhere.^37−39^

**Figure 3:**
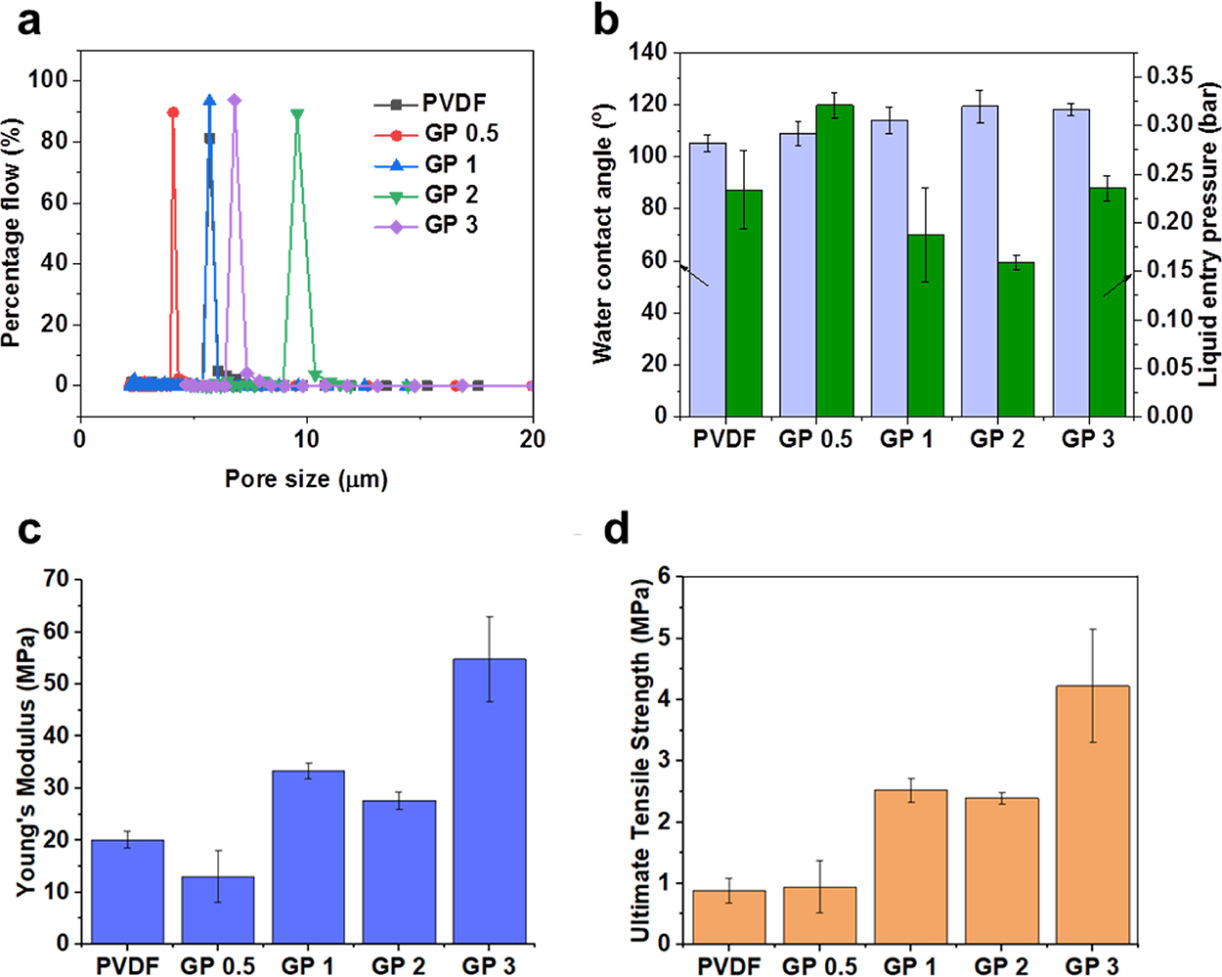
Morphological, wetting, and mechanical properties of the electrospun membranes including pore size distribution (a), water contact angle and liquid entry pressure (b), Young’s modulus (c), and ultimate tensile strength (d). Error bars represent standard deviations from three samples (or five in the case of water contact angle).

Capillary flow porometry revealed the electrospun membranes to have surprisingly narrow pore size distributions, as shown by representative plots in Figure 3a. In all cases, over 85% of the total gas flow detected during the measurement corresponded to pores of mean size. When looking at the pore size range (i.e., the biggest minus the smallest) and dividing this by the mean pore size for each membrane, the values are 1.006, 0.533, 0.305, 0.199, 0.177, and 0.179 for PTFE, PVDF, GP 0.5, GP 1, GP 2, and GP 3, respectively. In other words, with respect to the mean pore size values, the distribution of pore sizes is narrower for the electrospun membranes than for the commercial PTFE membranes. This is an important property of MD membranes since large pore size distributions will contain many pores that are either too big (and so risk becoming wetted) or too small (and unnecessarily hindering vapor transport).

In addition to narrow pore size distributions, these electrospun membranes have incredibly high porosities of around 90%. Table 1 summarizes the porosity values as well as other characteristics of these membranes. In general, higher membrane porosity results in higher permeability and flux values as there is more free volume in which the permeating species can travel. Typical phase inversion membranes have porosities in the range of 60–80%.^40^

**Table 1:** Summary of Membrane Properties and Performance Characteristics

membrane code	thickness (μm)	pore size: smallest; mean; largest (μm)	porosity (%)	N_2_ permeability (barrer)	flux (L m^–2^ h^–1^)^a^	permeate conductivity (μS cm^–1^),^a^ salt rejection (%)	permeate As^5+^ conc. (ppb)^a^^,^^b^
PTFE (commercial)	190 (±15)	0.26 (±0.02); 0.26 (±0.01); 0.40 (±0.09)	80 (reported)	1.75 × 10^8^ (±3.50 × 10^6^)	14.15 (±2.12)	1.8 (±0.2), >99.9	<0.045; <0.045^d^
PVDF	89 (±10)	5.69 (±0.03); 5.91 (±0.01); 7.48 (±0.25)	91.2 (±0.6)	1.21 × 10^9^ (±8.57 × 10^8^)	22.99 (± 1.06)	1.1 (±0.1), >99.9	<0.045
GP 0.5	68 (±29)	4.06 (±0.16); 4.20 (±0.19); 4.89 (±0.24)	87.6 (±1.8)	1.15 × 10^9^ (±1.54 × 10^8^)	22.87 (±1.33)	1.8 (±0.4), >99.9	<0.045
GP 1	69 (±26)	5.47 (±0.57); 5.65 (±0.59); 6.47 (±0.54)	87.8 (±0.4)	2.02 × 10^9^ (±4.36 × 10^8^)	20.99 (±5.46)	1.7 (±0.5), >99.9	<0.045
GP 2	70 (±14)	9.37 (±0.62); 9.80 (±0.73); 10.56 (±1.05)	91.9 (±0.4)	2.82 × 10^9^ (±3.06 × 10^8^)	27.94 (±1.77), 28.30^c^	2.1 (±0.2), 1.8^c^; >99.9	<0.045
GP 3	88 (±21)	6.11 (±0.71); 6.34 (±0.76); 7.25 (±0.08)	89.9 (±0.9)	1.70 × 10^9^ (±1.24 × 10^8^)	16.98 (±0.00)	2.0 (±0.1), >99.9	<0.045

a These values were measured after 24 h of continuous testing.

b 0.045 ppb represents the detection limit of the ICP-MS method.

c These values were measured after 5 days of continuous testing.

d This value corresponds to measured As^3+^ levels using feed water containing 300 ppb sodium meta-arsenite, also below the detection limit indicating perfect rejection of As^3+^.

It is therefore very promising to be able to fabricate membranes with significantly higher porosities while retaining sufficient mechanical properties to withstand handling and high-shear testing environments. The highest porosity value belonged to GP 2 with a value of 91.9 (±0.4%) after hot-pressing. This is higher than most nanofiber membranes found in the literature, which typically suffer reductions in porosity to below 90% due to post-treatment.^41−45^

The mechanical properties of the membranes, as elucidated by tensile testing, are presented in Figure 3c,d. A generally positive trend can be seen for both the ultimate tensile strength (UTS) and Young’s modulus (YM) values as the loading of POSS-rGO increased. There is no significant difference between these values for pure PVDF and GP 0.5, indicating a limited effect at such low loadings as 0.5 wt %. However, at 2 wt % (GP 2), the membrane exhibited a 38% increase in Young’s modulus and a 271% increase in ultimate tensile strength compared to the pure polymer. This increased further for the membrane with a loading of 3 wt % (GP 3), which exhibited a 479% increase in UTS with a value of 4.2 ± 0.9 MPa and a 272% increase in YM compared to the pure polymer. This represents more than a 2-fold increase in strength compared to materials with comparable porosity found in the literature.^46−49^ These changes are likely due to the attractive interactions between the PVDF polymer chains and the branched alkyl groups extending from the silica core of the POSS molecule. In addition, the high surface area and high intrinsic strength of the graphene basal plane provide strong interfacial interaction with the polymer, increasing both the strength and stiffness of the membranes.^50,51^ As seen from the SEM images (Figure 1c), some graphene flakes are located at the intersections between multiple nanofibers. This is another advantage of using 2D graphene as opposed to 1D materials like carbon nanotubes,^52^ which may enhance the strength of individual fibers but not necessarily the interconnections between fibers. It is at these weak interconnections where mechanical failure is most likely to occur, prompting researchers to try and fuse them together with methods such as solvent vapor treatment.^53^ In this case, however, the moderate hot-press treatment and the inclusion of POSS-rGO were sufficient to produce thin, highly porous, yet robust membranes. One other possibility is that the high thermal conductivity of graphene could have resulted in local concentrations of heat in regions where the POSS-rGO was in contact with the polymer nanofibers. This may have resulted in greater softening and fusing of the polymer in and around those regions, further increasing the mechanical strength after cooling. Further investigation is needed to prove this, however.

The wetting properties of the membranes were characterized by water contact angle measurements and liquid entry pressure (LEP) measurements. These are presented together in Figure 3c. The increased loading of POSS-rGO resulted in an increase in the water contact angle from 105 ± 3° for pure PVDF to 119 ± 6° for GP 2. This increase came despite the fact that the mean pore size of GP 2 was almost twice that of the PVDF membrane, as summarized in Table 1. Larger pore sizes tend to reduce the contact angle on hydrophobic surfaces such as PVDF as there is less material supporting the surface tension of the water droplet. In this case, however, the addition of the highly hydrophobic POSS-rGO counteracted this tendency and resulted in larger pores and a higher contact angle. GP 3, despite having a higher loading of POSS-rGO and smaller mean pore size than GP 2, had approximately the same contact angle value of 118 ± 2°. This could be due to agglomeration of the graphene at such high loadings resulting in a less homogeneous dispersion throughout the membrane. This would effectively reduce the available surface area of the hydrophobic graphene, resulting in a lower contact angle value than expected.

The liquid entry pressure values do not follow the same trend as the water contact angle values but do relate to the maximum pore size values for the membranes. The membrane with the smallest LEP was GP 2 with a value of 0.159 ± 0.007 bar. This membrane also had the largest maximum pore size value of 10.56 ± 1.05 μm, more than twice that of GP 0.5, which had the largest LEP value of 0.321 ± 0.013 bar. The remaining three membranes have very similar maximum pore size values, and their LEP values lie within one standard deviation of each other, indicating the link between maximum pore size and liquid entry pressure. Intuitively, the largest pore in a membrane is the one that requires the least amount of pressure to force liquid through, all else being equal. It is important to note that these LEP values are considerably lower than that of the commercial PTFE (3.683 ± 1.677 bar). This is due to the high intrinsic hydrophobicity of PTFE compared to PVDF but also the significantly smaller maximum pore size value of 0.40 (±0.09) μm. These low LEP levels did not seem to affect the ability of these membranes to achieve high salt rejection in membrane distillation experiments, as the following section highlights.

### Membrane Distillation Performance

**Figure 4:**
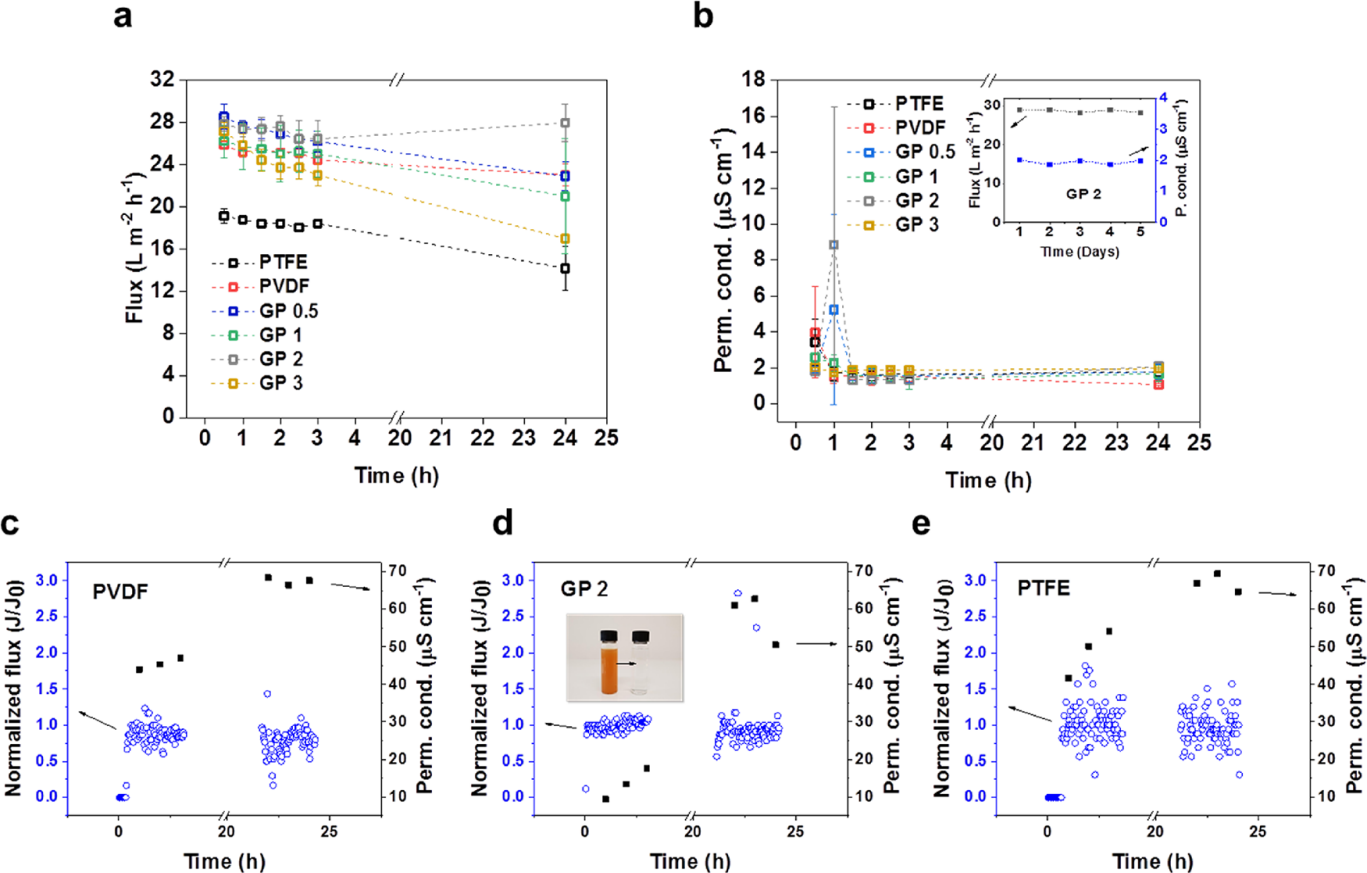
Summary of the flux (a) and permeate conductivity (b) data for the electrospun membranes and the commercial PTFE membrane where error bars represent the standard deviation from three different membranes. The inset in panel (b) is flux and permeate conductivity data for the 5 day continuous MD experiment using membrane GP 2. Panels (c–e) are normalized flux and permeate conductivity values from 24 h MD experiments using the feed solution with added calcium carbonate (10 mg L^–1^) and iron sulfate heptahydrate (2 g L^–1^) as foulants. The inset in panel (d) is a photograph of the feed solution and the permeate solution from the GP 2 membrane, showing the removal of color.

The flux and permeate conductivity values from the MD experiments are summarized in Figure 4a,b. A commercial PTFE membrane was chosen for comparison as they are generally considered to be the best-performing among current commercially available membranes in terms of flux, rejection, and energy efficiency.^54^ Most of the membranes, including the commercial PTFE, show a similar flux pattern over 24 h. A slight decline (<5%) in flux is observed over the first 3 h for all membranes except GP 0.5 and GP 3, which showed declines of 9.1 and 15.1%, respectively. This gradual decline continued over 24 h of testing except in the case of GP 2, whose flux was stable at a value of 27.94 ± 1.77 L m^–2^ h^–1^. This was 21.5% higher than the pure PVDF membrane nearly double that of the commercial PTFE membrane after the same time period. This increased flux can be largely attributed to the increased hydrophobicity and larger mean pore size of this membrane compared to others, as shown in Table 1. The N_2_ permeability for GP 2 was 57% higher than the pure PVDF membrane despite their porosities being almost identical. Larger pore sizes are known to reduce the resistance to mass transfer in MD but increase the risk of pore wetting.^55^ In this case, the high hydrophobicity of the membrane successfully prevented wetting despite its mean pore size value of 9.80 ± 0.73 μm being 1–2 orders of magnitude larger than is typical for MD membranes. It is possible that the membrane thickness affected the flux performance, particularly with respect to the PTFE membrane, which was more than twice as thick as the electrospun membranes. However, the difference in thickness between the electrospun membranes is not particularly significant. Furthermore, previous work has suggested that the membrane thickness plays a much less significant role in increasing the mass transfer resistance than the air gap, which is orders of magnitude thicker.^56−58^ In order to further test the flux stability of this membrane (GP 2), a 5 day continuous MD experiment was conducted, yielding a final flux value of 28.30 L m^–2^ h^–1^ and a corresponding permeate conductivity value of 1.786 μS cm^–1^. This is evidence of the high stability of the separation process for this type of feed solution. The more severe flux decline observed for the GP 3 membrane may be the result of increased temperature polarization due to the high thermal conductivity of the POSS-rGO. While at the lower loadings, this is unlikely to contribute significantly to temperature polarization; at 3 wt %, this property of the graphene may become detrimental to MD performance.

In general, all electrospun membranes produced very high quality permeates with conductivities of less than 2 μS cm^–1^. This corresponds to very high salt rejection values of >99.9%. More importantly, the arsenic levels in the permeate for all membranes, including PTFE, were below the detection limit of ICP-MS (<0.045 ppb). This means that all samples produced water of significantly higher quality than recommended by the WHO (<10 ppb). Though not tested using the electrospun membranes, the air gap membrane distillation (AGMD) system with the commercial PTFE was also effective in removing As^3+^ from a 300 ppb sodium meta-arsenite solution. In this case again, the arsenic levels were below the detection limit. High As rejections using commercial MD membranes have been also reported in the literature.^59^

### Inorganic Fouling Experiments

In order to test the membranes under fouling conditions, 10 mg L^–1^ calcium carbonate and 2 g L^–1^ iron sulfate heptahydrate were added to the feed solution to create near-saturation conditions. Upon dissolving, the ions will dissociate and recombine to form precipitates once the saturation limit has been reached. Two well-known inorganic foulants in membrane distillation are calcium sulfate and calcium carbonate as they become less soluble at higher temperatures. Previous studies have shown that these crystals can form on the membrane surface, eventually leading to pore blocking and flux decline.^25,60^ The normalized flux values for the pure PVDF, GP 2, and commercial PTFE membrane were plotted as a function of time to assess the flux stability in these harsh conditions.

As can be seen in Figure 4c–e, the flux values were fairly stable throughout the 24 h experiments for both electrospun and commercial PTFE membranes, although the PTFE membrane exhibited greater fluctuations throughout the test. There is a noticeable difference in the permeate conductivity for the first 3 h of testing for the GP 2 membrane, which was considerably lower than for the pure PVDF and PTFE membranes (9–20 μS cm^–1^ compared with 40–60 μS cm^–1^). However, after 24 h of testing, the permeate conductivities increased for all membranes to between 60 and 70 μS cm^–1^ for the PVDF and PTFE membranes and 50–63 μS cm^–1^ for GP 2, owing to the very high solute concentration resulting in partial wetting, which in turn enabled transport of inorganic solutes across the membrane. This lower permeate conductivity for the GP 2 membrane suggests that it had slightly better wetting resistance than the other two membranes but in all cases, the permeate quality is still very high and well within the range for safe drinking. Another difference between the membranes is the variability in the normalized flux values. For the PTFE membrane, the data points are highly scattered, showing rapid variation in flux values, whereas the PVDF values were more narrowly distributed and the GP 2 values more still. This rapid variability could be due to the presence of precipitates in the feed solution causing temporary pore blocking and then being removed due to the shear forces from the feed flow. It may be expected to affect the PTFE membrane more because the pores are significantly smaller and so may be more easily blocked. Nevertheless, overall, the flux was pretty stable for the membranes. The normalized flux values were averaged (excluding outliers) for the beginning and end of each test. The percentage differences of these values were −10.4, −8.7, and −6.4% for the PVDF, GP 2, and PTFE membranes, respectively. In other words, the average flux decline was highest for the PVDF membrane, then GP 2, and then the commercial PTFE membrane.

During the periods where the permeate was externally collected rather than recirculated, the feed water became more concentrated to the point where crystals were clearly visible in the water and on the surfaces of the feed vessel and tubing. This precipitation away from the membrane surface may explain the relatively stable flux values observed in these experiments. Once precipitation was initiated in the vessel, subsequent precipitation and crystal growth were favored there rather than on the membrane surface. This phenomenon has been reported before.^61^ Despite this, at the end of the experiments, the membranes were removed and though thoroughly rinsed in DI water and still had visible coloration from the feed water, suggesting that some precipitation did indeed occur on the membrane surface. This is further evidenced by the observed increases in permeate conductivity and the EDX data depicted in Figure S2. Pronounced peaks are observed for carbon and fluorine from all three membranes, as expected given their chemical makeup. In addition, the elements Fe and O are also prominent in the EDX spectra and overlap in the element maps. This is suggestive of iron oxides precipitating on the membrane, which are responsible for the red/brown color of the feed solution. The spectrum for the GP 2 membrane shows the presence of silicon, which originates from the POSS functional group on the graphene. Trace amounts of silicon detected on the PTFE membrane are likely due to contamination, and similarly, trace amounts of copper present in the electrospun membranes may be the result of contamination from the stainless steel collector plate on which the membranes were formed. Small peaks corresponding to sulfur are present on the electrospun membranes, again suggesting some precipitation of sulfate crystals on the membrane, although this peak is missing for the PTFE membrane, suggesting that precipitation occurred preferentially away from the membrane surface. Furthermore, the absence of calcium peaks from all spectra indicates that no CaCO_3_ crystals precipitated on the membrane but instead remained in the vessel or tubing. Similarly, the absence of an arsenic peak suggested that the arsenic was retained in the feed solution and did not adsorb onto the membrane in detectable quantities. The presence of platinum is a result of the membrane coating process during the sample preparation.

In the case of the electrospun membranes, the crystals that did precipitate on their surfaces did not grow sufficiently large to completely cover the pores, whereas the PTFE membrane has a much more densely coated surface. This may be the reason why the normalized flux values were much less stable for the PTFE membrane and may be an advantage of the electrospun architecture in more severe cases of inorganic fouling. Future work will involve longer-term experiments with various process conditions to see how best to control inorganic fouling to enable continuous operation.

Finally, by the end of the experiments, the feed volume had decreased from 1600 to approximately 600 mL – a reduction of 62.5%. This could have been continued, but this was the limit imposed by the geometry of the feed vessel. This highly effective concentration is useful in the context of arsenic removal where the discharge of brine into the local environment is highly dangerous.

## Conclusions

In conclusion, air gap membrane distillation experiments showed perfect rejection of arsenic from simulated groundwater of the Tacna region, Peru. High-performance electrospun PVDF membranes were enhanced in terms of mechanical properties, hydrophobicity, and membrane distillation performance with the addition of hydrophobic POSS-functionalized graphene. The optimal loading was 2 wt % with respect to the polymer, which resulted in a 280% increase in the ultimate tensile strength compared to the pure PVDF membrane. This membrane demonstrated a stable flux of ∼28 L m^–2^ h^–1^ over 5 days of continuous testing, while the pure PVDF membrane showed a 10.9% flux decline over just 24 h. This best-performing membrane had nearly twice the flux of a commercial PTFE membrane, but all membranes in all cases showed very high (>99.9%) rejection of salt, highlighting the effectiveness of air gap membrane distillation in removing inorganic contaminants. These membranes also performed well when treating a highly concentrated solution containing calcium carbonate, iron sulfate, sodium chloride, and sodium arsenate dibasic heptahydrate. The POSS-rGO membrane demonstrated more stable flux over 24 h of testing than the pure PVDF membrane and also higher rejection values. Fouling with iron oxides was evident from EDX measurements but seemed to be more prominent on the commercial PTFE membrane due to the crystal size being comparable to or greater than the pore size. The much larger pore sizes of the electrospun membranes meant that they were not blocked or covered by the foulant crystals. Minimal amounts of sulfur and no traces of calcium were found from the EDX measurements, which suggest that AGMD may be a suitable technology for zero liquid discharge applications. In the case of treating arsenic-contaminated groundwater, this approach is necessary in order to prevent further environmental damage.

## Materials and Methods

### Materials

For the functionalization reaction, GO (1 wt % aqueous suspension) was purchased from William Blythe (Lancashire, UK), aminopropyl isobutyl polyhedral oligomeric silsesquioxane (AM0265 – referred to here as POSS) was purchased in powder form from Hybrid Plastics (USA), and *N*,*N*′-dicyclohexylcarbodiimide (DCC) and tetrahydrofuran (THF) were purchased from Sigma Aldrich (Germany). Electrospinning solutions were prepared using polyvinylidene difluoride (PVDF - *M*_w_ = 534,000 g mol^–1^) and *N*,*N*-dimethylformamide (DMF), both purchased from Sigma Aldrich, Germany, as well as acetone (Fisher Scientific, UK). Millipore deionized (DI) water (18 MΩ cm resistivity) was used for the preparation of feed solutions along with sodium arsenate dibasic heptahydrate and sodium meta-arsenite, which were purchased from Sigma Aldrich. NaCl, CaCO_3_, and FeSO_4_·7H_2_O also used for the feed solutions were purchased from Acros, Belgium. All reagents and materials were used as received.

### Graphene Oxide Functionalization

Functionalization of GO with POSS occurred via amide formation, following the same method described elsewhere.^22^ Briefly, 100 mg of GO was freeze-dried from an aqueous suspension using liquid nitrogen. The dried GO was then redispersed in 50 mL of THF in a sonication bath (Elmasonic, 80 kHz frequency at 100% power) for 2 h. This was then decanted into a 250 mL round bottom flask along with 2 g of POSS and 100 mg of DCC. This mixture was sonicated for a further 10 min and then refluxed at 80 °C for 48 h. Following this, the remaining solvent was evaporated and the powder was heat-treated at 120 °C for 8 h to partially reduce the GO. The powder was then redispersed in 50 mL of THF, poured into approximately 500 mL of methanol, and then filtered using a homemade polyacrylonitrile filter (0.2 μm pore size). This last step was repeated three times to remove any unreacted POSS, and the powder (POSS-rGO) was then placed in a vacuum oven at 80 °C and then stored for further use. To investigate the chemical changes further, the whole procedure was repeated without the heat treatment step removed. This yielded POSS-GO (note that this is now nonreduced GO), which was characterized for comparison purposes but not used for the preparation of membranes. The reaction scheme for the functionalization of GO with POSS can be found in Figure S4.

### Fabrication of Electrospinning Solutions

The electrospinning polymer solutions were prepared by dissolving 1.4 g of PVDF powder in 8.6 g of a DMF/acetone mixture with a ratio of 1:2, making solutions with a total weight of 10 g in each case. This solvent mixture contained various quantities of POSS-rGO as described in Table S2 in the Supporting Information. This was done by first producing a 20 mg mL^–1^ solution of POSS-rGO in DMF via sonication followed by the addition of acetone and a final step of stirring overnight at 40 °C until the polymer completely dissolved.

### Fabrication of Electrospun Membranes

Electrospun membranes were prepared using a setup that consisted of a syringe pump (Cole Parmer), a high voltage supply, and a stainless steel tray, which was used as a collector. Prior to spinning, the dope solutions were individually drawn into a 10 mL plastic syringe (BD Emerald), which was left standing on end for a few minutes to allow any bubbles to escape. Then a 19G 1.1 × 50 mm needle (BD Microbalance) whose sharp end was flattened by abrading it with sand paper was fixed to the syringe. This was then clamped onto the syringe pump, and the needle was connected to the high voltage supply using a crocodile clip. The collector plate with an area of 552 cm^2^ was connected to the opposite terminal of the high voltage supply, again using a crocodile clip, and was placed 20 cm from the tip of the needle.

Once the syringe needle and collectors were connected, the program on the syringe pump was run, and the high voltage supply was switched on. The voltage and dope solution flow rate were kept constant for each dope solution at 18 kV and 5 mL h^–1^, respectively.

After the dope solution was deposited, the membrane was left to dry overnight under a fume hood. The membrane was then carefully peeled off the collector plate and placed flat on a 250 × 230 mm sheet of tempered glass. An identical piece of glass weighing 785.2 g was placed on top of the membrane, exerting a pressure of 13.94 N m^–2^. This was then placed in an oven at 170 °C, just below the melting temperature of PVDF, for 1 h in order to compact the fibers and increase the mechanical stability of the membrane. After this post-treatment, the membrane was removed and stored for further use.

### Characterization of Functionalized Graphene Oxide

#### X-ray Photoelectron Spectroscopy

The functionalization of GO with POSS was assessed by X-ray photoelectron spectroscopy (XPS) using an Axis Ultra spectrometer (Kratos Analytical Limited, Manchester, UK) with a monochromatic Al Kα source (1486.7 eV). The spectra were analyzed using CasaXPS software.

#### Attenuated Total Reflectance Fourier Transform Infrared Spectroscopy

In addition, attenuated total reflectance Fourier transform infrared spectroscopy (ATR-FTIR) was used to probe the GO functionalization. This was carried out using an iDS Nicolet iS5 spectrometer (Thermo Scientific, UK), with a Ge crystal as a background. The wavenumber range was 650–4000 cm^–1^, and the step size was 0.5 cm^–1^.

### Characterization of Electrospun Membranes

#### Scanning Electron Microscopy

The membranes were imaged using scanning electron microscopy (SEM) (QUANTA FEI 200, USA) with a 15 kV acceleration voltage and a 2.5 mm spot size. To prepare the samples, small pieces of each membrane were stuck onto SEM holders using carbon tape and were sputtered with gold (or platinum for the fouled membranes) with a layer thickness of 5–6 nm to render the samples electrically conductive.

#### Energy-Dispersive X-ray Spectroscopy

In conjunction with SEM imaging, elemental analysis of the membranes was performed with EDX spectroscopy. This was used to analyze the components from the inorganic fouling experiments, and spectra were collected using an Oxford Instruments X-Max detector and plotted using AZtec 3.3 SP1software.

#### Tensile Testing

The mechanical properties of the membranes were investigated by tensile testing. Measurements were carried out using an Instron 5542 tensiometer (Instron, USA) with a 100 N load cell under ambient conditions. Samples were prepared by cutting rectangular strips of membranes (7 mm × 60 mm) and sandwiching each end between two 10 mm squares of thin cardboard using double-sided sticky tape. The effective length of each sample was 40 mm, giving a length/width ratio of 5.71:1. Three identical samples were prepared for each membrane. The thickness of the membranes was measured with a digital micrometer screw gauge in proximity to where the tensile strips were cut. The tensile strips themselves were not measured as the compression from the micrometer may have affected the mechanical properties or induced a defect. Ten thickness measurements were taken for each membrane and averaged. The elongation rate was set up to 10 mm min^–1^, and ultimate tensile strength and Young’s modulus values were calculated.

#### Capillary Flow Porometry

The pore size distributions and N_2_ permeability of the electrospun membranes were measured by capillary flow porometry (Porolux 1000, POROMETER, Belgium). This employed the gas–liquid displacement method using perfluoropolyether (Porefil 125, surface tension = 15.88 ± 0.03 mN m^–1^) as the wetting liquid as detailed in our previous work.^62^ The slope of the dry curve was used to calculate the nitrogen permeability by dividing by the membrane thickness. This technique was also used to measure the liquid entry pressure (LEP) of the membranes. Using a nonstandard method, 13 mm disks were cut from each membrane and inserted dry into the Porolux device. Then, 0.3 mL of DI water was dropped onto the surface of the membrane, and the compartment was closed by connecting the gas. The Porolux was set to provide a maximum pressure of 1 bar over 50 steps, and the “full porometry” program was executed. This gradually increased the pressure on the water sat atop the membrane. As the pressure continued to increase, a sudden increase in gas flow was measured by the device, indicating that the water was forced through the membrane. The pressure at which this occurred was reported as the LEP. For all measurements, the reported values are averages of three samples taken from different areas on the membrane.

#### Porosity

Membrane porosity, ε, was evaluated using the gravimetric method, as reported previously.^35,62^ Briefly, 10 mm squares were cut out of the membranes (3 for each membrane) and weighed. Then these squares were immersed in the same liquid used for porometry (Porefil 125) for 30 s to become fully wetted. One by one, the squares were removed from the wetting liquid and placed on tissue paper and were gently daubed, removing any residue from the surfaces. The samples were then weighed again in order to determine the mass of wetting liquid, which was adsorbed by the pores. The membrane porosity was then calculated using
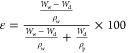
1where *W*_w_ is the wet membrane weight and *W*_d_ is the dry membrane weight. The densities of Porefil 125 (ρ_w_) and the PVDF polymer (ρ_p_) are 1.9 and 1.78 g cm^–3^, respectively. The values reported were the averages of three measurements.

#### Water Contact Angle

The wetting properties of the membranes were evaluated using water contact angle (CA) measurements as described previously.^63^ Membrane strips were fixed to glass slides, which were then placed on the stage of an Attension Theta optical tensiometer, and five drops were measured for each membrane and averaged.

#### Water Quality Analysis

The quality of the permeate produced from membrane distillation experiments was assessed in terms of conductivity using a Fisher Scientific Accumet XL200 conductivity meter. In addition, As^3+^ and As^5+^ were quantified using inductively coupled plasma mass spectroscopy (ICP-MS, Agilent 7700x) and Masshunter Version 5 software.

#### Membrane Distillation Tests

Arsenic removal experiments were performed using air gap membrane distillation, as depicted in Figure S5. The system is composed of two isolated water loops – one containing tap water used for cooling the condenser plate inside the membrane module and one containing the heated feed water. The prepared synthetic solutions had concentrations of inorganic arsenic and sodium chloride similar to the concentrations of arsenic and conductivity recorded in water sources intended for human consumption in the rural area of the city of Tacna, Peru (Locumba River and Sama River). The feed water contained 600 ppb sodium arsenate dibasic heptahydrate and sufficient NaCl to bring the feed conductivity up to 2500 μS cm^–1^ – similar to that of the Locumba river. To prepare the feed solutions, a concentrated (5 mg/L) stock solution was prepared by dissolving 10 mg of sodium arsenate dibasic heptahydrate in 500 mL of DI water. Then, 60 mL of this stock solution was added to 1940 mL of DI water to produce 2 L of feed solution. Tests were carried out using a feed water volume of 1 L. A test was also conducted on the commercial PTFE membrane to see if As^3+^ could be removed by AGMD. For this, a 300 ppb solution of sodium meta-arsenite was prepared instead of sodium arsenate dibasic heptahydrate by diluting a 1000 mg L^–1^ stock solution.

After prior optimization, the process conditions were selected, as summarized in Table S3 in the Supporting Information. The permeate samples were collected in a measuring cylinder after 1 h of conditioning for each membrane. The flux was calculated by extrapolating the volume of permeate collected over 30 min, given a membrane area of 27.33 cm^2^, and the salt rejection was calculated from permeate conductivity values, as described previously.^56^ For the inorganic fouling experiments, the normalized flux was calculated using
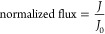
2as the ratio of the flux at a particular time to the initial flux (measured after 1 h of conditioning, as before).

#### Inorganic Fouling Experiments

In order to test the membranes’ propensity for inorganic fouling, 10 mg L^–1^ calcium carbonate and 2 g L^–1^ iron(III) sulfate heptahydrate were added to the same arsenic and sodium chloride feed solution used for prior experiments. This turned the water a terracotta color as shown in Figure 4d (inset). These contaminants, among others, are present in the water within the Tacna region of Peru and have been shown to cause significant fouling issues in various membrane applications.^26,64,65^ In these experiments, the mass of the permeate was measured using a weighing scale (Adam Highland HCB 3001) connected to a data logger collecting one data point every 2 min in order to track any sudden changes in flux. The permeate was collected for 3 h at the beginning and then was recirculated overnight and collected again for 22, 23, and 24 h of the 24 h experiment, during which time the loss of the permeate resulted in the increased concentration of the feed – the aim being to reach saturation conditions. At each hour of permeate collection, a sample was taken for conductivity measurements and then returned to the collection vessel. All other process conditions were kept the same.

## Supporting Information Available

Experimental details for functionalization of graphene oxide with aminopropyl isobutyl-POSS and membrane preparation syntheses as well as membrane characterization and membrane distillation conditions (PDF)

## Abbreviations

AGMD: air gap membrane distillation
ATR-FTIR: attenuated total reflectance Fourier transform infrared spectroscopy
CA: contact angle
DCC: *N*,*N*′-dicyclohexylcarbodiimide
DI: deionized
DMF: dimethyl formamide
EDX: energy-dispersive X-ray spectroscopy
GO: graphene oxide
ICP-MS: inductively coupled plasma mass spectrometry
LEP: liquid entry pressure
MD: membrane distillation
SEM: scanning electron microscopy
POSS: polyhedral oligomeric silsesquioxane
POSS-rGO: polyhedral oligomeric silsesquioxane-reduced graphene oxide
PTFE: polytetrafluoroethylene
PVDF: polyvinylidene difluoride
THF: tetrahydrofuran
UTS: ultimate tensile strength
WHO: World Health Organization
XPS: X-ray photoelectron spectroscopy
YM: Young’s modulus
