# Supplementary material for: POSS-Functionalized Graphene Oxide/PVDF Electrospun Membranes for Complete Arsenic Removal Using Membrane Distillation
Source: ACS Appl Polym Mater. 2021 Mar 5;3(4):1854–65. doi: 10.1021/acsapm.0c01402 (PMC8154216; doi:10.1021/acsapm.0c01402)
Supplement: Supplementary file 1 — ap0c01402_si_001.pdf [file ap0c01402_si_001.pdf]

Supplementary Information

POSS-functionalized graphene oxide/PVDF electrospun membranes for complete arsenic removal using membrane distillation

*Sebastian Leaper[a], Edgardo Oscar Avendaño Cáceres[b], Jose Miguel Luque-Alled[a], Sarah H. Cartmell[c], Patricia Gorgojo[a] **

[a] Department of Chemical Engineering and Analytical Science, School of Engineering, The University of Manchester, Manchester, M13 9PL, UK

[b] Faculty of Engineering, Universidad Nacional Jorge Grohmann, Avenida Miraflores S/N, Miraflores 23000, Peru

[c] Department of Materials, School of Natural Sciences, The University of Manchester, Manchester, M13 9PL, UK

* Email address: p.gorgojo@manchester.ac.uk

Characterization Results

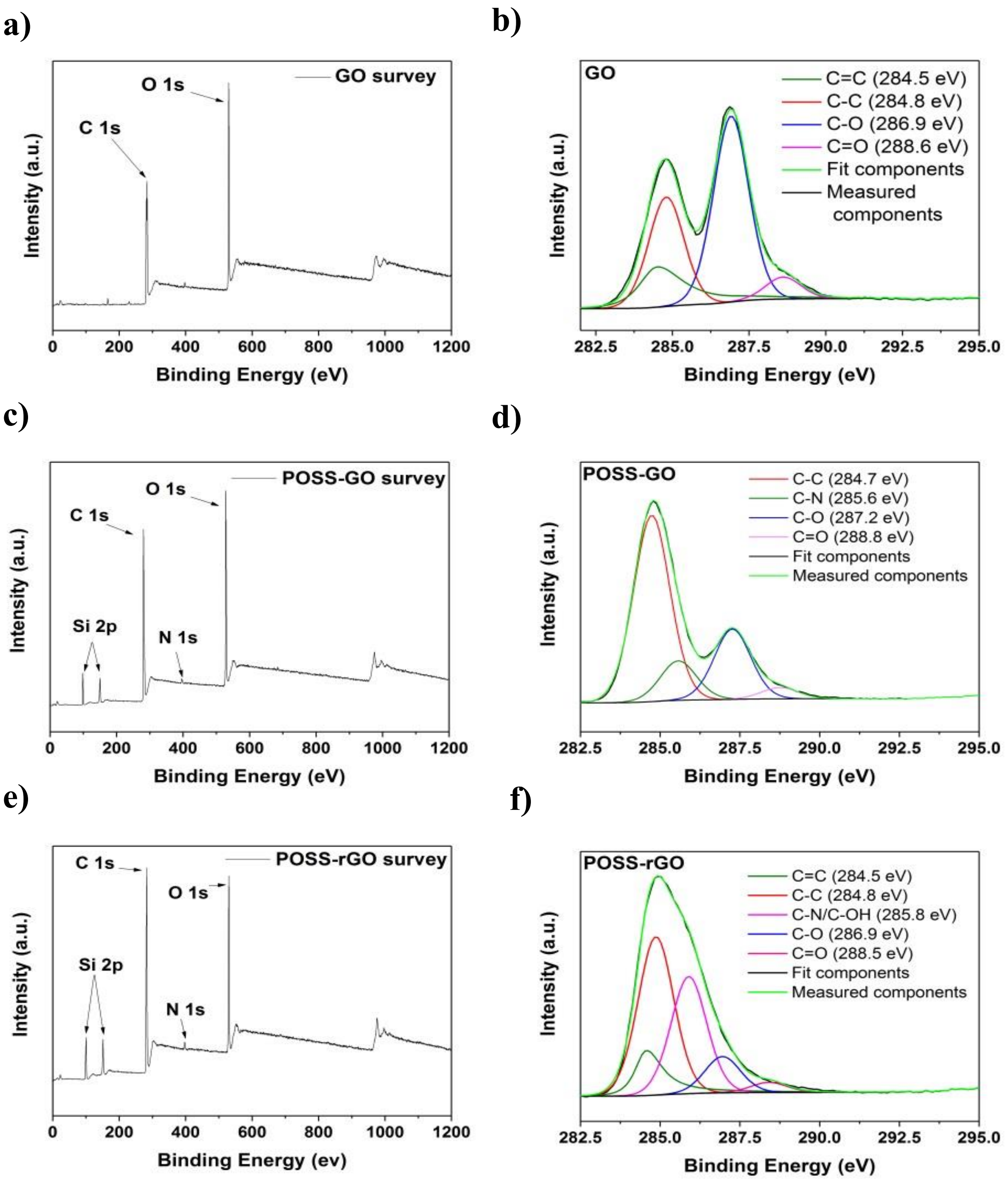

Figure S1. X-ray photoelectron spectroscopy surveys and C 1s high resolution spectra for GO (a & b), POSS-GO (c & d) and POSS-rGO (e & f), respectively.

Table S1. Ratios of elements in GO and POSS-functionalized GO (reduced and non-reduced) from XPS measurements.

	Percentage (%)		
**Component**	**GO**	**POSS-GO**	**POSS-rGO**
C	69.69	65.44	64.39
O	30.31	21.77	17.67
N	-	1.17	1.55
Si	-	11.62	16.39

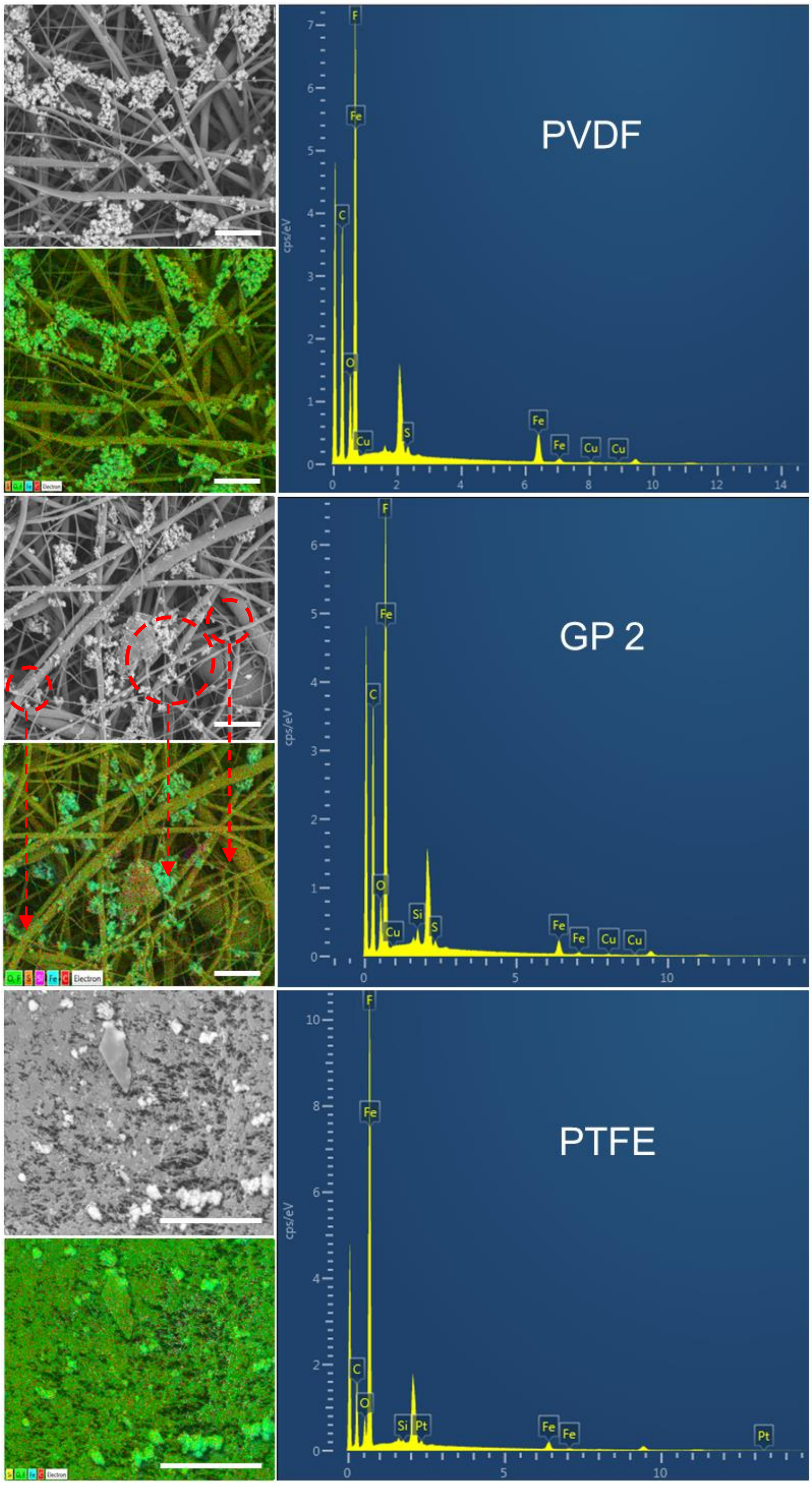

Figure S2. Scanning electron micrographs with X-ray dispersive spectroscopy maps and spectra for the PVDF and GP 2 electrospun membranes and the commercial PTFE membrane taken after the inorganic fouling tests. A pronounced Si peak in the GP 2 image corresponds to the POSS-rGO. This can be seen in the purple clusters on the surface of the graphene flakes highlighted by the red dashed circles. The scale bars represent 10 µm.

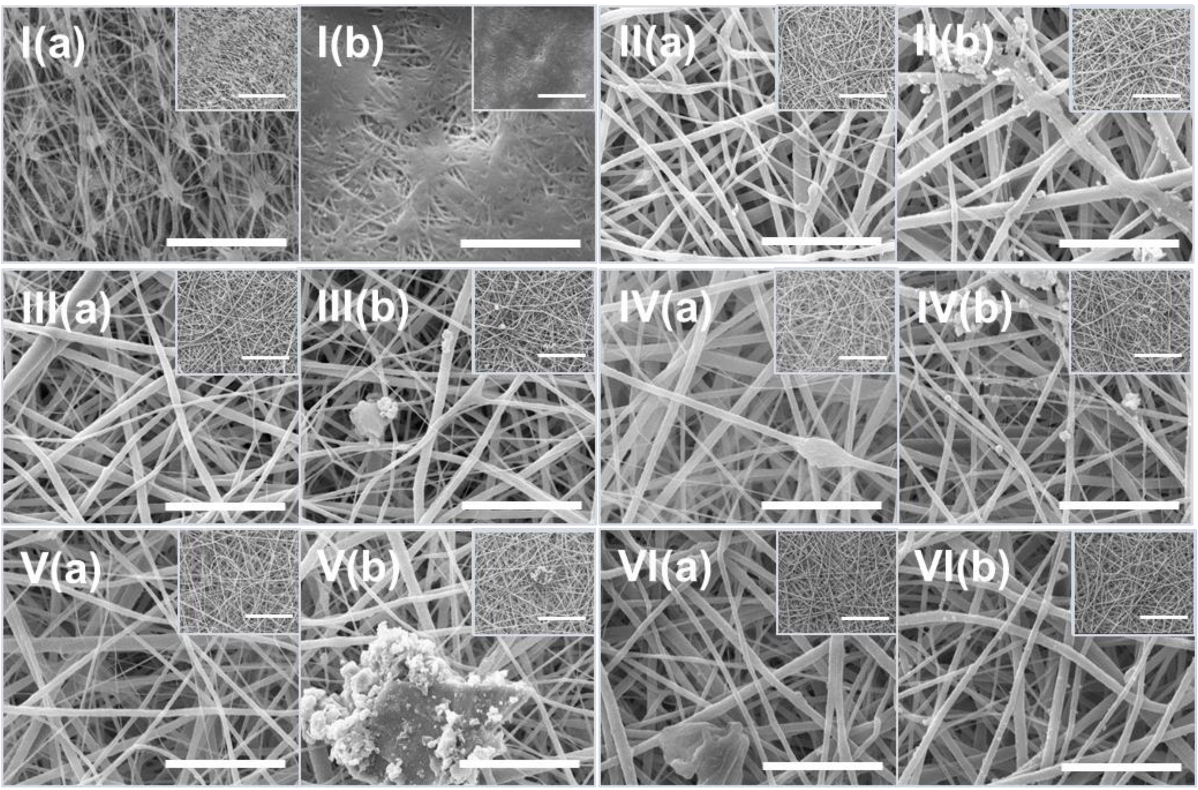

Figure S3. Scanning electron microscope images of all electrospun membranes before (a) and after (b) membrane distillation experiments. The numbers correspond to the membranes as follows: I) commercial PTFE; II) pure PVDF electrospun membrane; III) GP 0.5; IV) GP 1; V) GP 2 and VI) GP 3. The scale bar on the large images represent 5 µm for PTFE (1 a & b) and 20 µm for the electrospun membranes and their magnifications are 12000x and 3000x, respectively. The inset images are at lower magnifications – 6000x for PTFE and 800x for the electrospun membranes and the scale bars represent 10 and 50 µm, respectively. Inset photographs in I(a) and VI(a) indicate the colour difference between the PVDF and GP 3 membranes, cut to a diameter of 2 cm.

Experimental Details

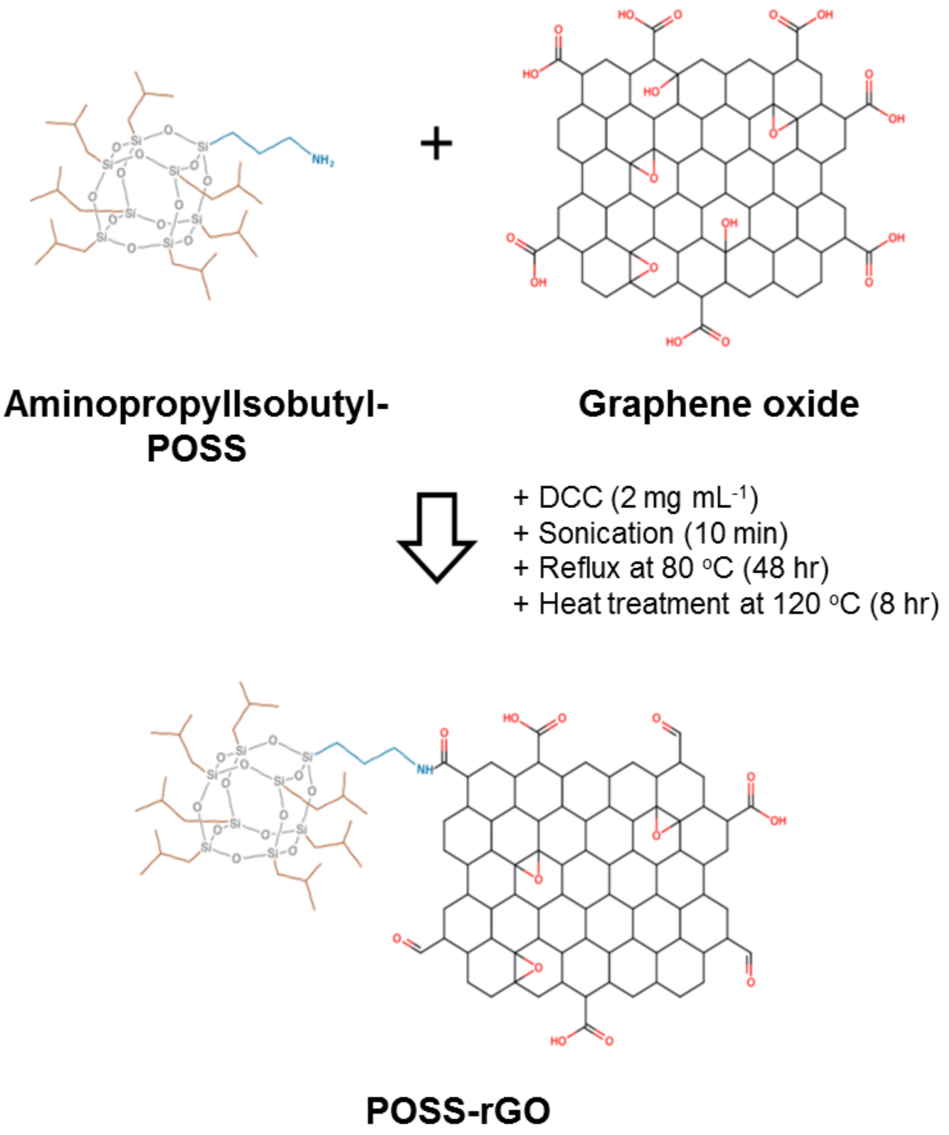

Figure S4. Reaction scheme for the functionalization of graphene oxide with aminopropyl isobutyl-POSS.

Table S2. A summary of the components in the electrospinning solutions.

Membrane code	Wt.% GO filler in casting solution	PVDF (g)	POSS-rGO (mg)	DMF (g)	Acetone (g)
PVDF	0	1.4	0	2.8667	5.733
GP 0.5	0.5	1.4	7	2.8597	5.733
GP 1	1	1.4	14	2.8527	5.733
GP 2	2	1.4	28	2.8387	5.733
GP 3	3	1.4	42	2.8107	5.733

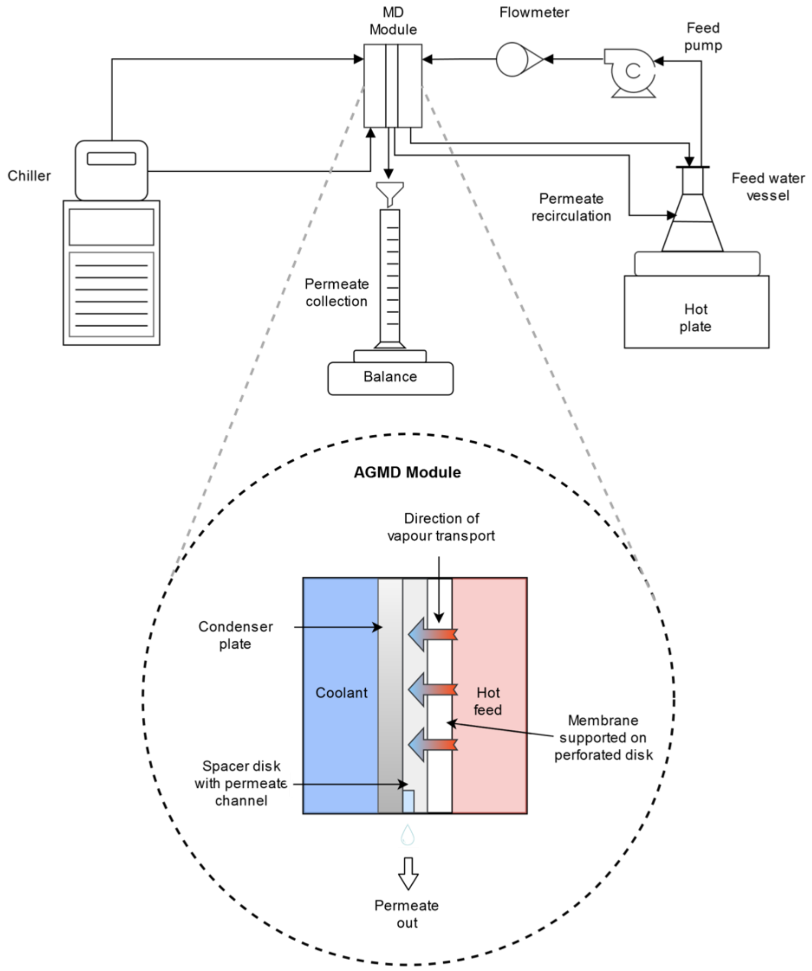

Figure S5. Schematic of the membrane distillation system used for arsenic removal. Feed water volumes were 1 L.

Table S3. A summary of the process conditions for the membrane distillation experiments.

**Configuration**	**Air gap width (mm)**	**Feed flow rate (mL min^{-1})**	**Feed temperature (^{o}C)**	**Coolant temperature (^{o}C)**
AGMD	3	750	80	20
